# Potential Mechanisms of the Protective Effects of the Cardiometabolic Drugs Type-2 Sodium–Glucose Transporter Inhibitors and Glucagon-like Peptide-1 Receptor Agonists in Heart Failure

**DOI:** 10.3390/ijms25052484

**Published:** 2024-02-20

**Authors:** Giovanna Gallo, Massimo Volpe

**Affiliations:** 1 Department of Clinical and Molecular Medicine, Sapienza University of Rome, Via di Grottarossa 1035–1039, 00189 Rome, Italy; giovanna.gallo@uniroma1.it; 2 IRCCS San Raffaele Roma, Via della Pisana 235, 00163 Rome, Italy

**Keywords:** heart failure, SGLT2i, GLP1-RA, cardiometabolic drugs, heart failure management

## Abstract

Different multifactorial pathophysiological processes are involved in the development of heart failure (HF), including neurohormonal dysfunction, the hypertrophy of cardiomyocytes, interstitial fibrosis, microvascular endothelial inflammation, pro-thrombotic states, oxidative stress, decreased nitric oxide (NO) bioavailability, energetic dysfunction, epicardial coronary artery lesions, coronary microvascular rarefaction and, finally, cardiac remodeling. While different pharmacological strategies have shown significant cardiovascular benefits in HF with reduced ejection fraction (HFrEF), there is a residual unmet need to fill the gap in terms of knowledge of mechanisms and efficacy in the outcomes of neurohormonal agents in HF with preserved ejection fraction (HFpEF). Recently, type-2 sodium–glucose transporter inhibitors (SGLT2i) have been shown to contribute to a significant reduction in the composite outcome of HF hospitalizations and cardiovascular mortality across the entire spectrum of ejection fraction. Moreover, glucagon-like peptide-1 receptor agonists (GLP1-RA) have demonstrated significant benefits in patients with high cardiovascular risk, excess body weight or obesity and HF, in particular HFpEF. In this review, we will discuss the biological pathways potentially involved in the action of SGLT2i and GLP1-RA, which may explain their effective roles in the treatment of HF, as well as the potential implications of the use of these agents, also in combination therapies with neurohormonal agents, in the clinical practice.

## 1. Introduction

Heart failure (HF) is a severe disease representing a dreadful pandemic affecting 64 million people worldwide, with a progressively growing prevalence destined to further rise as a consequence of the improved prognosis of the diseases causing this syndrome, as well as the aging of the population [1,2]. About a half of patients present HF with preserved ejection fraction (EF) (HFpEF, EF ≥ 50%), with a risk of death and hospitalization comparable to subjects with reduced EF (HFrEF, EF < 40%) [2]. Among the different risk factors and comorbidities associated with the development of HFpEF, hypertension, diabetes, age, chronic kidney disease (CKD) and obesity play a fundamental role [3,4,5,6,7,8].

It has been described that these conditions promote complex and multifactorial pathophysiological processes which lead to the hypertrophy of cardiomyocytes, interstitial fibrosis, microvascular endothelial inflammation, a pro-thrombotic state, oxidative stress, decreased nitric oxide (NO) bioavailability, energetic dysfunction, epicardial coronary artery lesions, coronary microvascular rarefaction and, finally, to increased left ventricular (LV) diastolic stiffness and filling pressures and impaired relaxation [9,10,11,12,13,14,15]. Moreover, preserved LVEF does not exclude systolic dysfunction, which may consist of reduced mid-wall shortening and impaired longitudinal strain, lower cardiac output, and higher peripheral resistances. In such a context, increased arterial stiffness associated with increased LV end-systolic elastance contributes to abnormalities in ventricular–arterial interaction, limiting cardiac output also in HFpEF [16,17,18]. Renin–angiotensin–aldosterone system (RAAS) activation induces vasoconstriction, cardiac hypertrophy, fibrosis, inflammation, vascular smooth muscle cell de-differentiation, and reactive oxygen species production. These actions, largely mediated through the action of the biological effector angiotensin II at the receptor levels, may promote structural remodeling, cardiac and vascular damage, and the development of HF [19,20].

On the basis of these premises, different studies and randomized clinical trials using different pharmacological strategies have been conducted in patients with HF.

In HFrEF, pharmacological agents acting on neurohormonal systems, such as angiotensin-converting enzyme inhibitors (ACEi), angiotensin receptor blockers (ARBs), mineralocorticoid receptor antagonists (MRAs), beta-blockers (BBs), and angiotensin receptor–neprilysin inhibitors (ARNi), have been shown to significantly reduce cardiovascular mortality and HF hospitalizations, thus being recommended as first-line strategies [21,22].

Conversely, these pharmacological strategies generally failed to demonstrate statistically significant benefits in HFpEF. However, the studies conducted in HFpEF barely missed statistical significance and left a consistent level of uncertainty, possibly in view of the wide heterogeneity of HFpEF phenotypes. On the other hand, some recent meta-analyses showed a reduction in HF hospitalizations and mortality with both RAAS inhibitors (RAASi) and the ARNi sacubitril/valsartan [23]. Pooled analyses including data from patients with HF with mildly reduced EF (HFmrEF; EF: 41–49%) also produced positive results [24,25,26]. Altogether, these observations had an impact on the most recent international guidelines on the management of HF [22]. Nonetheless, there is a residual unmet need to fill the gap in terms of knowledge of mechanisms and efficacy in the outcomes of neurohormonal agents in HFpEF. In particular, it cannot be excluded that these drugs may work better in specific HFpEF phenotypes.

More recently, type-2 sodium–glucose transporter inhibitors (SGLT2i) have shown a significant reduction in the composite outcome of HF hospitalizations and cardiovascular mortality in HFpEF, and this observation has been received with enthusiasm by the scientific community. In particular, empagliflozin and dapagliflozin have been viewed as disease-modifying drugs and immediately positioned in the management of HFpEF by international guidelines [22]. The mechanisms underlying the favorable effects of SGLT2i in HFpEF (as well as in HFrEF) are still unclear, though many reasonable hypotheses have been advanced.

On the other hand, another class of drugs initially proposed for the management of type 2 diabetes, glucagon-like peptide-1 receptor agonists (GLP1-RA), has demonstrated significant benefits in patients with excess body weight or obesity and HFpEF [27]. In fact, semaglutide at the dosage of 2.4 mg s.c. per week improved exercise performance and quality of life in HFpEF patients [28] and reduced by 18% the composite outcome of cardiovascular death and HF hospitalizations in patients with overweight or obesity without diabetes [29]. The recently concluded SOUL (Semaglutide cardiOvascular oUtcomes triaL) study will provide results about the effects of oral semaglutide on the risk of cardiovascular events in individuals with type 2 diabetes and established atherosclerotic cardiovascular disease and/or chronic kidney disease [30].

**Figure 1:**
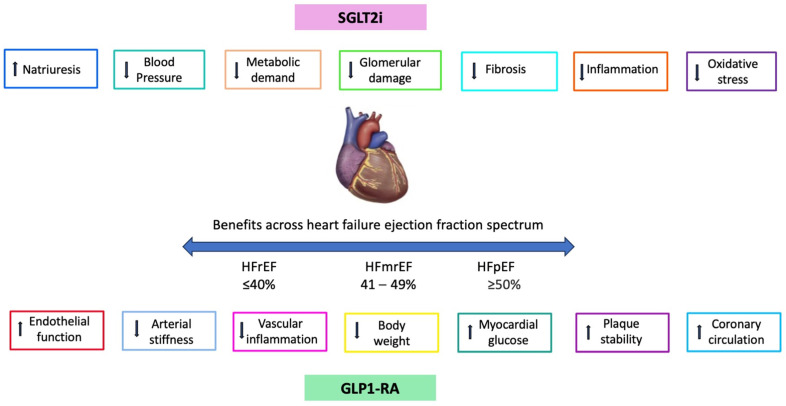
Mechanisms potentially underlying early and long-term effects in heart failure across the whole ejection fraction spectrum. Upward arrows stand for increase. Downward arrows stand for decrease.

The aim of this review is to discuss the biological pathways potentially involved in the action of SGLT2i and GLP1-RA which may explain their effective role in the treatment of HF (Figure 1), as well as the potential implications of the use of these agents, also in combination therapies with neurohormonal agents, in clinical practice.

## 2. Mechanisms of SGLT2i Benefits in Heart Failure

The main mechanism of action of SGLT2i consists of the reduction in sodium–glucose reabsorption in the proximal convoluted tubule, resulting in glycosuria, natriuresis, and diuresis. In addition, these compounds promote the reduction in filtration pressure, the restoration of the normal production of filtrate, and the decrease in renal oxygen demand. Other important effects related to SGLT2i include reductions in oxidative stress, apoptosis, tubular–interstitial inflammation, and fibrosis and improvements in mitochondrial function and autophagy [31,32,33]. Indeed, a restoration of the tubular–glomerular feedback loop occurs, since the macula densa senses the increased sodium levels leading to the vasoconstriction of the afferent arterioles and the simultaneous vasodilation of efferent arterioles [34].

**Figure 2:**
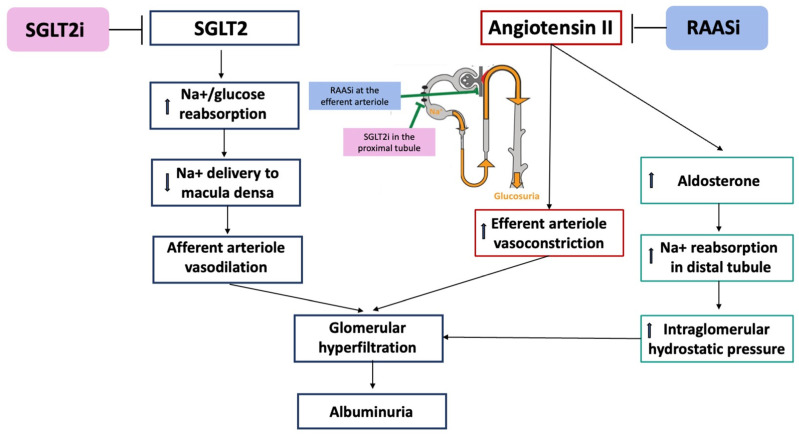
Synergistic action of RAASi and SGLT2i at the tubulo-glomerular level. Upward arrows stand for increase. Downward arrows stand for decrease.

The combination of SGLT2i and RAASi has been associated with additive cardiovascular- and nephro-protective effects, consisting of greater vasodilation; osmotic diuresis; weight loss; and reductions in systemic oxidative stress, inflammation, blood pressure, glomerular injury, renal fibrosis, and proteinuria [35]. The clinical data available at this time do not rule out the hypothesis of a synergistic action of RAASi and SGLT2i, especially at the tubulo-glomerular level, where they both act on Na+ handling and natriuresis (Figure 2).

Preclinical studies have demonstrated that SGLT2i exert protective effects against renal ischemia–reperfusion injury, reducing the secretion of proinflammatory cytokines and reactive oxygen species and improving renal structure and function [36]. At the cardiac level, SGLT2i decrease calmodulin-kinase II activity with an improvement in sarcoplasmic reticulum calcium (Ca2þ) flux and contractility. SGLT2i also inhibit the Na/H^+^ exchanger and mammalian target of rapamycin, modulating cardiac autophagy and lysosomal degradation [37,38].

Throughout their osmotic and diuretic effects and the modulation of sympathetic nervous system activity, SGLT2i are also associated with blood pressure-lowering actions, thus contributing to lower cardiac afterload to improve ventricular arterial coupling and cardiac efficiency, to reduce LV mass index, and to promote cardiac reverse remodeling [39]. Moreover, SGLT2i attenuate endothelial cell activation and induce vasorelaxation through the activation of protein kinase G and voltage-gated potassium channels, with these mechanisms being associated with favorable hemodynamic effects [40]. It has also been proposed that SGLT2i induce a shift from the sympathetic to parasympathetic nervous system at baroreceptors’ level. In a study performed in 825 patients with both hypertension and diabetes, empagliflozin compared to a placebo produced a greater systolic blood pressure (SBP) reduction of 3.44 mmHg and 4.16 mmHg at the dosages of 10 mg and 25 mg, respectively [41]. Diastolic BP (DBP) was reduced by 1.36 mmHg and 1.72 mmHg with 10 mg and 25 mg of empagliflozin, respectively. Empagliflozin also produced a significant decrease in seated office SBP and DBP [41].

The SACRA (SGLT2i and Angiotensin Receptor Blocker Combination Therapy in Patients with Diabetes and Uncontrolled Nocturnal Hypertension) study demonstrated a significant reduction in office SBP (−7.9 mmHg and −4.2 mmHg in patients younger and older than 75 years, respectively) and 24 h SBP (−11.0 mmHg and −8.7 mmHg in patients younger and older than 75 years, respectively) in the group treated with empagliflozin compared to a placebo [42]. Consistent results were obtained in a placebo-controlled trial conducted in 311 patients, in which 12 weeks of treatment with 10 mg of dapagliflozin reduced office SBP and 24 h SBP by 4.28 mmHg and 4.45 mmHg, respectively [43]. In addition, a post hoc analysis of the study showed that SGLT2i had a synergistic BP-lowering effect with calcium channel blockers and beta-blockers. These antihypertensive effects persisted at the 52-week follow-up [43]. Also, canagliflozin has demonstrated BP-lowering effects, producing significant reductions in SBP (−4.0 mmHg and −4.8 mmHg at the dosages of 100 and 300 mg, respectively), 24 h SBP (−3.3 mmHg and −4.9 mmHg at the dosages of 100 and 300 mg, respectively) and DBP (−1.9 mmHg and −2.9 mmHg at the dosages of 100 and 300 mg, respectively) [44]. A post hoc analysis of the CREDENCE (Canagliflozin and Renal Events in Diabetes with Established Nephropathy Clinical Evaluation) trial showed a BP reduction by 3.5 mmHg compared to baseline in patients who received canagliflozin. In small studies, SGLT2i were associated with a significant reduction in LV mass index and LV filling pressure and with the improvement in LV diastolic function assessed using a tissue Doppler, with these effects being maintained after 12 months of therapy [45].

With regard to the effects on cardiac metabolism, SGLT2i metabolize adipose tissue fatty acids and increase circulating ketone levels, which represent a more efficient source of energy and an extra source of “fuel” and have been suggested to improve cardiac energetics and efficiency [46]. SGLT2i have also been shown to target inflammatory pathways independently from glucose-lowering effects. SGLT2i inhibit the nucleotide-binding domain-like receptor protein inflammasome (in particular, nucleotide-binding oligomerization domain, leucine rich repeat, and pyrin domain-containing 3 [NLRP3]), reduce the number of proinflammatory macrophagic M1 cells, and increase the number of anti-inflammatory M2 polarized cells [47]. Moreover, SGLT2i reduce the accumulation and inflammation of perivascular adipose tissue, which promote fibrosis and coronary artery disease through the secretion of leptin, tumor necrosis factor-a, and plasminogen activator inhibitor-1 [48]. In addition, SGLT2i have been demonstrated to increase erythropoietin secretion, which may exert favorable effects on cardiomyocyte mitochondrial function and myocardial tissue oxygen delivery [49].

In the EMPEROR-Reduced (Empagliflozin Outcome Trial in Patients with Chronic Heart Failure and a Reduced Ejection Fraction) [50] and DAPA-HF (Dapagliflozin and Prevention of Adverse Outcomes in Heart Failure) [51] studies, empagliflozin and dapagliflozin, respectively, produced a 25% reduction in the composite outcome of cardiovascular mortality and HF hospitalizations in patients with HFrEF. These benefits were evident from the first weeks of treatment, suggesting a potential influence of blood pressure reduction and of diuresis on the achieved results.

Besides their recognized role as a pillar treatment in HFrEF, the benefits of SGLT2i have been indeed confirmed and extended across the whole spectrum of LVEF. The EMPEROR-Preserved (Empagliflozin Outcome Trial in Patients with Chronic Heart Failure with Preserved Ejection Fraction) study showed a 21% reduction in the primary composite endpoint of HF hospitalizations and cardiovascular death in patients treated with empagliflozin compared to a placebo, with these findings being evident already starting from 18 days of treatment and being maintained over time [52]. Empagliflozin also slowed the decline in renal function and improved quality of life at 52 weeks. These findings were consistent in the group of patients with LVEF > 50%, in both males and females, and in patients with and without type 2 diabetes [52]. Consistently, in the DELIVER (Dapagliflozin Evaluation to Improve the Lives of Patients with Preserved Ejection Fraction Heart Failure) study, dapagliflozin reduced the primary composite outcome of worsening HF or cardiovascular mortality by 18%. A combined pooled analysis of both trials confirmed these results and reinforced the evidence of an SGLT2i class-effect in HFpEF [53].

In addition, the SOLOIST-WHF (Effect of Sotagliflozin on Cardiovascular Events in Patients with Type 2 Diabetes Post Worsening Heart Failure) study investigated the effects of sotagliflozin in diabetic patients with recently worsening HF, showing a 33% reduction in HF hospitalizations and cardiovascular death at 9-month follow-up independently from LVEF and renal function [54].

More recently, the EMPULSE (A Study to Test the Effect of Empagliflozin in Patients Who Are in Hospital for Acute Heart Failure) study explored the efficacy of empagliflozin in 530 patients with acute de novo or decompensated HF [55]. Empagliflozin reduced the primary outcome, defined as a hierarchical composite of all-cause death, total HF events, time to first HF event, or a ≥5-point change from baseline in the Kansas City Cardiomyopathy Questionnaire (KCCQ) total symptom score, independently from LVEF, diabetes, and onset time of HF and without significant adverse events compared to the placebo [55]. Potential explanations of the SGLT2i effects in acute HF derive from the results of the EMPAG-HF (Empagliflozin in Acute Decompensated Heart Failure) study, in which 60 patients were randomized to receive empagliflozin or a placebo in addition to standard therapy within 12 h of admission [56]. Empagliflozin produced a 25% greater total urine output without influence on the estimated glomerular filtration rate (eGFR), serum (creatinine, urea, cystatin-C), or urinary (total protein, albumin, α1-microglobulin) markers of renal function and injury [56].

A meta-analysis including 20,241 patients with HFrEF and HFpEF demonstrated that SGLT2i reduced all-cause and cardiovascular mortality (−14%), the composite of cardiovascular mortality, HF hospitalizations, or urgent visits for HF (−25%) independently from sex, age, eGFR, New York Heart Association (NYHA) class, LVEF, and diabetes [57].

## 3. Mechanisms of GLP1-RA Benefits in Cardiovascular Diseases

In the last few years, GLP1-RA, including short-acting molecules (exenatide, liraglutide, and lixisenatide) and long-lasting second-generation molecules (semaglutide, exenatide LAR, albiglutide, and dulaglutide), have been demonstrated to exert different protective cardiovascular actions and to represent effective body weight reduction strategies for overweight and obese patients [58,59,60,61].

GLP1 receptors are expressed in endothelial cells, vascular smooth muscle cells, macrophages, and monocytes, supporting an anti-atherosclerotic effect of GLP1-RAs [62,63,64]. Indeed, GLP1-RAs decrease reactive oxygen species (ROS) production in endothelial cells and cardiomyocytes and reduce circulating levels of 8-iso prostaglandin and the accumulation of monocytes/macrophages in the vascular wall. GLP1-RAs also inhibit the expression of adhesion molecules, such as vascular cell adhesion molecule-1 (VCAM-1), monocyte chemotactic protein-1 (MCP-1), E-selectin, and intercellular adhesion molecule-1 (ICAM-1) and their transformation in foam cells, slowing the process of atherosclerotic plaque formation [65,66]. Moreover, GLP1-RAs lead to endothelial-dependent vasorelaxation, inducing the expression of endothelial NO synthase (eNOS) while reducing endothelin levels [67].

Preclinical studies have demonstrated that GLP1-RA administration reduces hepatic proprotein convertase subtilisin/kexin type 9 (PCSK9), induces low-density lipoprotein receptor (LDLR) expression, and suppresses postprandial triglycerides and chylomicron secretion. In diabetic patients, GLP1-RAs have been shown to attenuate postprandial ApoB48 production and the clearance of triglyceride-rich chylomicrons [68].

Animal models have shown that GLP1-RA slow plaque progression, preserve the integrity of the fibrous cap, reduce plaque hemorrhage, and thus prevent plaque rupture [69].

Other studies have also demonstrated the prevention of the accumulation of epicardial adipose tissue, which contributes to the secretion of proinflammatory adipokines and to coronary atherosclerosis progression [70].

GLP1 circulating levels have been shown to rapidly increase during acute ST elevation and non-ST elevation myocardial infarction (MI) and are correlated with a composite outcome of recurrence of non-fatal MI, stroke, and cardiovascular death [71]. In addition, GLP1 levels on admission predicted 30-day outcomes better than other cardiac biomarkers such as high-sensitivity troponin T, C-reactive protein, and N-terminal pro-brain natriuretic peptide (NT-proBNP) [71]. An anti-ischemia–reperfusion injury action of GLP1-RA has been also proposed, but it is still unclear whether this effect is maintained in subjects already treated with this pharmacological class.

GLP1-RA also reduce RAAS activation, thus playing a BP-lowering role and protecting from the development of albuminuria, end-stage renal disease, and from renal-related deaths [72]. The genetic attenuation of the expression of GLP1 receptors has been associated with angiotensin II-induced hypertension and with the attenuation of vasoprotective and BP-lowering effects of liraglutide [73].

In such a context, different studies have shown a reduction in major cardiovascular events (MACE) in patients treated with GLP1-RA. In the LEADER (Effects of Liraglutide on Cardiovascular Outcomes in Patients With Diabetes With or Without Heart Failure) study, patients treated with liraglutide had a significantly lower risk of a composite outcome of cardiovascular death, nonfatal myocardial infarction, or nonfatal stroke independently from HF. Liraglutide produced a 13% reduction in HF risk, although not reaching statistical significance [74]. Consistent results were obtained with subcutaneous semaglutide in the SUSTAIN-6 (Trial to Evaluate Cardiovascular and Other Long Term Outcomes with Semaglutide in Subjects with Type 2 Diabetes) study [75] and with dulaglutide in the REWIND (Researching Cardiovascular Events with a Weekly Incretin in Diabetes) trial [76]. The PIONEER-6 (Oral Semaglutide and Cardiovascular Outcomes in Patients with Type 2 Diabetes) study showed the non-inferiority of oral semaglutide compared to a placebo, but it was not powered for MACE superiority and had a short follow-up [77]. A meta-analysis including about 56,000 subjects showed that GLP1-RA reduced the risk of MACE by 12%, of all-cause mortality by 12%, of renal disease progression by 17%, and of HF hospitalizations by 9% [78].

However, little evidence is available about the role of GLP1-RA in HF prevention and progression.

In the EXSCEL (Effects of Once-Weekly Exenatide on Cardiovascular Outcomes in Type 2 Diabetes) study, no significant effects of exenatide were observed in the subgroup of HF patients (2389 out of 14,752) with regard to all-cause mortality and to the composite of all-cause mortality and HF hospitalization, while exenatide significantly reduced these endpoints in subjects without HF [79]. Also, in the SUSTAIN-6 study, semaglutide did not significantly reduce MACE in patients with HF [75]. In the LEADER trial, baseline HF did not influence the achieved results [74].

The LIVE (Effect of Liraglutide on Left Ventricular Function in Stable Chronic Heart Failure Patients) study conducted in 241 patients with HFrEF showed no significant effects of liraglutide on LVEF, quality of life, or functional class after 24 weeks [80]. The post hoc analysis of biomarkers evidenced a 27% and 25% reduction in levels of mid-regional pro-atrial natriuretic peptide (MRproANP) and NT-proBNP, respectively [80]. The FIGHT (Functional Impact of GLP-1 for HF Treatment) study, which included 300 patients with recently decompensated HFrEF, did not show a superiority of liraglutide over the placebo on HF-related outcomes or functional capacity [81].

In a smaller study of 82 HFrEF patients, albiglutide was not able to improve LVEF, BNP levels, the 6 min walk test, myocardial glucose, or oxygen use after 13 weeks [82].

The HARMONY trial, conducted in 309 diabetic patients, investigated HF hospitalizations as an exploratory endpoint, showing a 29% reduction in the group treated with albiglutide compared to the placebo [83].

GLP1-RA, in particular liraglutide, have shown positive effects on LV diastolic function as a possible consequence of the improvement in cardiometabolic dysregulation, myocardial hypertrophy, fibrosis, and LV global longitudinal strain and of the reduction in atrial and ventricular volume [84,85].

In the STEP-HFpEF study, 529 patients with HFpEF and body-mass indexes ≥30 Kg/m^2^ were randomized to receive once-weekly semaglutide (2.4 mg) or a placebo for 52 weeks. Subjects treated with semaglutide experienced a significant decrease from baseline in the KCCQ clinical summary score, a 10.7% body weight reduction, and a 20.3 m improvement in 6 min walk distance, without significant differences in the rate of adverse events [28]. The ongoing SUMMIT study is investigating the effects of tirzepatide in obese patients with HFpEF [86].

Table 1 summarizes the main available studies exploring the effects of SGLT2i and GLP1-RA on cardiovascular endpoints.

**Figure 3:**
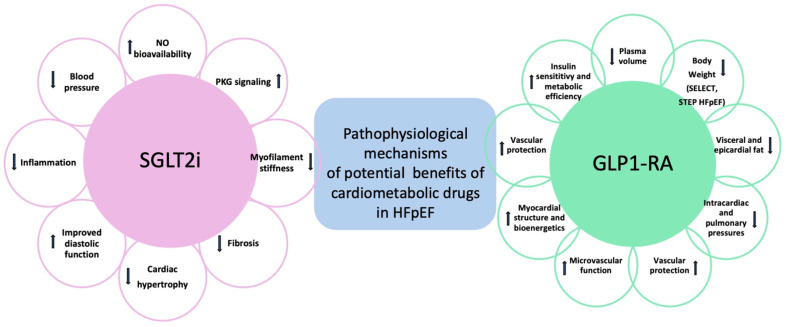
Pathophysiololgical mechanisms of potential benefits of SGLT2i and GLP1-RA in HFpEF. Upward arrows stand for increase. Downward arrows stand for decrease.

Figure 3 describes the pathophysiological mechanisms of potential benefits of SGLT2i and GLP1-RA in HFpEF.

## 4. Comparison of SGLT2i vs. GLP1-RA

No head-to-head trial has compared the effects of SGLT2i and GLP1-RA on MACE, and the available evidence is limited to real-word studies and meta-analyses.

In a meta-analysis including 77,242 patients, GLP1-RA reduced the risk of MACE by 12% and SGLT2i by 11% with a comparable magnitude, with this treatment effect being restricted only to subjects with established atherosclerotic cardiovascular disease [87]. SGLT2i reduced hospitalizations for HF by 31%, whereas GLP1-RA did not have a significant effect. Both GLP1-RA and SGLT2i reduced the progression of kidney disease, but only SGLT2i decreased worsening eGFR, end-stage kidney disease, and renal death [87].

In a study which enrolled about 41,500 patients, GLP1-RA were associated with a reduced risk of non-fatal MI, a composite of all cause-death, non-fatal MI, non-fatal stroke, and stable angina compared to SGLT2i, while no difference was detected in the incidence of HF hospitalization and stroke between the two groups. Similar results were found in the subgroup of patients without previous cardiovascular diseases [88].

Another study including 12,375 individuals showed that the risk of MACE was similar among patients treated with SGLT2i or GLP1-RA, whereas the risk of HF was 20% lower in the SGLT2i group, although without significant statistical difference. No significant interactions were observed across subgroups of age; sex; eGFR; HbA1c; HF; or the use of RAASi, insulin, or lipid-lowering drugs [89].

An analysis of the Swedish Diabetes registry also reported a similar risk of MACE, cardiovascular death and MI in patients treated with SGLT2i or GLP1-RA, but with an increased risk of stroke for SGLT2i [90]. No differences between empagliflozin and liraglutide were found with regard to the risk of MACE, HF hospitalizations, and all-cause mortality in a Danish registry-based study [90].

No studies have been specifically performed with the aim to investigate the potential synergistic actions of SGLT2i and GLP1-RA in the prevention and treatment of HF. Indeed, both these pharmacological agents modulate BP, inflammation, endothelial function, and cardiac fibrosis [91].

On the basis of the cardiovascular protective role of SGLT2i and GLP1-RA, the European Association for the Study of Diabetes, the American Diabetes Association [92], and the American College of Cardiology [93] recommend considering these pharmacological classes as add-on therapies in patients with type 2 diabetes and atherosclerosis disease, preferring SGLT2i in patients with history of HF or at high risk of developing HF. The American Association of Clinical Endocrinologists suggest prescribing SGLT2i or GLP1-RA in high-cardiovascular-risk patients independent of glycemic levels [94]. The European Society of Cardiology guidelines recommend both SGLT2i and GLP1-RA as first-line therapy in diabetic patients at high cardiovascular risk. SGLT2i should be prescribed as first-line therapy in HF patients independently from LVEF [95].

Although no trials have specifically examined the effects of combining SGLT2i with other recommended HF treatments (RAASi, ARNi, BBs, and MRAs), the recent STRONG-HF (Safety, Tolerability, and Efficacy of Rapid Optimization, Helped by NT-proBNP Testing, of Heart Failure Therapies) study has demonstrated that a quick uptitration of guideline-directed medical therapy under strict follow-up significantly reduces the outcome of HF readmission and all-cause mortality [96]. These results suggest that the different pharmacological classes act additively and that the patients should be protected with all these treatments as soon as possible if tolerated [97].

## 5. Conclusions

HF, in particular HFpEF, is a complex syndrome with different underlying pathophysiological mechanisms and multiple phenotypic and clinical expressions. The heterogeneous phenotypes and clinical presentations lead to cardiac hypertrophy, fibrosis, and oxidative stress in which LV diastolic dysfunction is the common disease expression sliding towards overt HF. In such a context, SGLT2i have been clearly demonstrated to exert several favorable cardiovascular effects which may contribute to reversing cardiac remodeling and to delaying the progression to overt HF. Also, GLP1-RA have shown very promising results in clinical trials performed in diabetic and/or overweight patients. Future studies are needed to investigate and confirm the potential benefits of the association of these pharmacological classes, which may represent a novel intriguing therapeutic strategy [98].

## Figures and Tables

**Table 1:** Summary of the main available studies exploring the effects of SGLT2i and GLP1-RA on cardiovascular endpoints.

Study	Study Design	Study Population	Results
EMPA-REG BP [41]	Randomized controlled trial Empagliflozin vs. placebo	825 patients with both hypertension and type 2 diabetes	Significantly greater reductions in SBP, DBP, and seated office SBP and DBP in the empagliflozin group
SACRA [42]	Randomized controlled trial Empagliflozin vs. placebo	132 patients with type 2 diabetes and uncontrolled nocturnal hypertension receiving stable antihypertensive therapy including ARBs	Significant reduction in office SBP and 24 h SBP in the empagliflozin group
Weber et al. [43]	Randomized controlled trial Dapagliflozin vs. placebo	311 patients with uncontrolled type 2 diabetes (HbA1c 70–105%) and hypertension (SBP 140–165 mmHg and DBP 85–105 mmHg) receiving oral antihyperglycemic drugs, insulin, or both, plus a RAAS blocker and an additional antihypertensive drug	Dapagliflozin (10 mg) significantly reduced office SBP and 24 h SBP with a synergistic BP-lowering effect with calcium channel blockers and beta-blockers
Pfeifer et al. [44]	Post hoc analysis canagliflozin vs. placebo	Pooled data from four 26-week, randomized, double-blind, placebo-controlled studies in patients with type 2 diabetes (n = 2313) and a 6-week, randomized, double-blind, placebo-controlled, ABPM study in patients with type 2 diabetes and hypertension (n = 169).	Canagliflozin significantly reduced SBP, 24 h SBP, and DBP
CREDENCE post hoc analysis [45]	Post hoc analysis canagliflozin vs. placebo	4401 patients with type 2 diabetes and CKD	Canagliflozin increased the likelihood of achieving a 30% reduction in UACR with a lower risk of kidney outcomes, MACEs and hospitalization for HF or cardiovascular death
EMPEROR-Reduced [50]	Randomized controlled trial empagliflozin vs. placebo	3730 patients with HFrEF	Empagliflozin reduced the composite outcome of cardiovascular death or hospitalization for worsening HF (−25%)
DAPA-HF [51]	Randomized controlled trial dapagliflozin vs. placebo	4744 patients with HFrEF	Dapagliflozin reduced the composite outcome of cardiovascular death or worsening HF (−26%)
EMPEROR-Preserved [52]	Randomized controlled trial empagliflozin vs. placebo	5988 HF patients with EF > 40%	Empagliflozin reduced the composite outcome of cardiovascular death or worsening HF (−21%)
DELIVER [53]	Randomized controlled trial dapagliflozin vs. placebo	6263 HF patients with EF > 40%	Dapagliflozin reduced the composite outcome of cardiovascular death or worsening HF (−18%)
SOLOIST-WHF [54]	Randomized controlled trial sotagliflozin vs. placebo	1222 HF patients with type 2 diabetes who were recently hospitalized for worsening HF	Sotagliflozin reduced the composite outcome of cardiovascular death or worsening HF (−33%)
EMPULSE [55]	Randomized controlled trial empagliflozin vs. placebo	530 patients with acute de novo or decompensated HF	Empagliflozin reduced the primary hierarchical composite outcome of all-cause death, total HF events, time to first HF event, or a ≥5-point change from KCCQ symptom score
EMPAG-HF [56]	Randomized controlled trial empagliflozin vs. placebo	60 patients hospitalized for acute decompensated HF	Addition of empagliflozin to standard medical treatment resulted in a 25% increase in cumulative urine output over 5 days without affecting markers of renal function or injury
Cardoso et al. [57]	Meta-analysis of placebo-controlled, randomized trials of SGLT2i in patients with HF	20,241 patients with HFrEF and HFpEF	SGLT2i reduced all-cause and cardiovascular mortality (−14%), the composite of cardiovascular mortality, HF hospitalizations, or urgent visits for HF (−25%)
LEADER [74]	Randomized controlled trial liraglutide vs. placebo	9340 patients with type 2 diabetes and high cardiovascular risk	Liraglutide reduced the risk of a composite outcome of cardiovascular death, nonfatal myocardial infarction, or nonfatal stroke independently from HF. Liraglutide produced a 13% reduction in HF risk, although not reaching statistical significance
SUSTAIN-6 [75]	Randomized controlled trial once-weekly subcutaneous semaglutide vs. placebo	3297 patients with type 2 diabetes	Semaglutide reduced the composite outcome of cardiovascular death, nonfatal myocardial infarction, or nonfatal stroke
REWIND [76]	Randomized controlled trial dulaglutide vs. placebo	9901 patients with type 2 diabetes at high cardiovascular risk with high HbA1c	Dulaglutide reduced the composite outcome of non-fatal myocardial infarction, non-fatal stroke, or death from cardiovascular causes
PIONEER-6 [77]	Randomized controlled trial oral semaglutide vs. placebo	3183 patients with type 2 diabetes at high cardiovascular risk	Semaglutide was not inferior compared to placebo in reducing MACEs (death from cardiovascular causes, nonfatal myocardial infarction, or nonfatal stroke)
EXSCEL [79]	Randomized controlled trial exenatide vs. placebo	14,752 diabetic patients, 2389 with HF	No significant effects of exenatide in the subgroup of HF patients with regard to all-cause mortality and to the composite of all-cause mortality and HF hospitalization. Significant effects in patients without HF
LIVE [80]	Randomized controlled trial liraglutide vs. placebo	241 patients with HFrEF	No significant effects of liraglutide on LVEF, quality of life, or functional class
FIGHT [81]	Randomized controlled trial liraglutide vs. placebo	300 patients with recently decompensated HFrEF	Liraglutide did not significantly reduce the primary endpoint of time to death, time to rehospitalization for HF, and time-averaged proportional change in NT-proBNP level
HARMONY [83]	Randomized controlled trial albiglutide vs. placebo	309 patients with type 2 diabetes	Albiglutide reduced the exploratory endpoint of HF hospitalizations by 29%
SELECT [29]	Randomized controlled trial subcutaneous once-weekly semaglutide vs. placebo	17,604 patients with pre-existing cardiovascular disease, with overweight or obesity, without diabetes	Semaglutide reduced the composite endpoint of death from cardiovascular causes, nonfatal myocardial infarction, or nonfatal stroke
STEP-HFpEF [28]	Randomized controlled trial subcutaneous once-weekly semaglutide vs. placebo	529 patients with HFpEF and obesity	Semaglutide reduced KCCQ clinical summary score and improved 6MWT distance

See text for abbreviations.

## Data Availability

No new data were created.
